# The Interplay of Politics and Conspiracy Theories in Shaping Vaccine Hesitancy in a Diverse Cultural Setting in Italy

**DOI:** 10.3390/ijerph22020230

**Published:** 2025-02-06

**Authors:** Christian J. Wiedermann, Barbara Plagg, Patrick Rina, Giuliano Piccoliori, Adolf Engl

**Affiliations:** Institute of General Practice and Public Health, College of Health Professions—Claudiana, 39100 Bolzano, Italy

**Keywords:** vaccine hesitancy, conspiracy theories, South Tyrol, public health, COVID-19, misinformation, cultural dynamics, political influence, grey literature

## Abstract

Vaccine hesitancy presents a significant challenge to public health, particularly in culturally diverse regions, such as South Tyrol, Italy. This article examines the interplay between political influences, conspiracy theories, and vaccine hesitancy in South Tyrol, an autonomous province characterised by its linguistic diversity and historical scepticism toward central authority. This study aimed to identify the important drivers of vaccine hesitancy and propose targeted strategies to enhance vaccine acceptance. Peer-reviewed and grey literature was examined to explore the sociocultural factors, political dynamics, and conspiracy narratives influencing vaccine hesitancy in South Tyrol. The analysis incorporated publicly available materials, including propaganda from anti-vaccine organisations, and regional public health data to contextualise the findings. Vaccine hesitancy in South Tyrol was influenced by historical tensions with the central government, cultural alignment with Austrian healthcare practices, and politically motivated opposition to vaccination. Conspiracy theories disseminated by local organisations and political entities exploit concerns regarding governmental overreach and personal autonomy. These dynamics are compounded by the selective misrepresentation of scientific discourse, which further polarises public opinion. Addressing vaccine hesitancy in South Tyrol requires culturally sensitive communication, community engagement through trusted local figures, transparency in health policies, and the proactive monitoring of misinformation. These strategies can mitigate mistrust and promote vaccine acceptance in regions with similar sociopolitical complexities.

## 1. Introduction

Vaccine hesitancy, defined as reluctance or refusal to vaccinate despite the availability of vaccines, poses a significant challenge to public health worldwide [1,2]. This phenomenon is influenced by a complex interplay of factors including political beliefs, cultural contexts, and the proliferation of conspiracy theories [3,4].

In regions characterised by distinct linguistic and cultural identities, such as South Tyrol, an autonomous province in northern Italy, the coexistence of diverse cultural groups within a limited geographical area presents unique challenges, as beliefs, attitudes, and values differ significantly among these groups and substantially influence public perceptions and behaviours regarding vaccination [5]. South Tyrol is characterised by its German-speaking majority and Italian-speaking minority. This linguistic diversity has historically influenced health information-seeking behaviours [6] and the trust in health authorities [7]. Studies indicate that COVID-19 vaccine hesitancy varies among linguistic groups, with German speakers aged 20–39 showing more hesitancy than Italian speakers [8]. No significant differences were found among older age groups. German speakers exhibited less trust in institutions and the media and reported lower well-being. Italians agreed more with the national vaccination plan and sought vaccine-related information more frequently than other groups, including Ladin speakers and those classified as ’other’. Significant hesitancy differences were also noted between German speakers and the ’other’ group, and between Italian speakers and the ’other’ group [8].

South Tyrol has a long-standing tradition of scepticism towards vaccinations, particularly within its German-speaking communities, where alternative medicine has historically played a central role in healthcare beliefs. This scepticism extends beyond COVID-19 vaccines and has influenced parental attitudes toward childhood immunisation, with many parents delaying or refusing vaccines due to concerns about ingredients, perceived over-medicalisation, and a belief in natural immunity [5]. The preference for natural health approaches and distrust in centralised health authorities—amplified by the province’s historical tensions with the Italian government—have contributed to persistent vaccine hesitancy across generations [9]. These factors highlight the cultural dimension of vaccine attitudes in South Tyrol and the challenge of increasing acceptance in a population that frequently accesses non-mainstream medical information.

A recent qualitative study on vaccine scepticism in South Tyrol revealed that vaccine-hesitant parents often question the necessity and timing of childhood vaccinations, particularly for non-mandatory vaccines perceived as unnecessary for young children. Parents’ decision-making is influenced by concerns about vaccine ingredients, potential side effects, and the preference for natural immunity. Scepticism is reinforced by the distrust in health authorities and perceived coercion in vaccine policies [10]. This context is crucial for understanding vaccine hesitancy in South Tyrol, where COVID-19 vaccination and routine childhood immunisation face considerable resistance [5].

The political landscape is a significant factor in the formation of vaccine attitudes. Political ideologies and affiliations may influence individuals’ trust in government-led health initiatives and their susceptibility to misinformation. Research suggests that individuals with populist or anti-establishment political orientations exhibit a higher propensity to endorse conspiracy theories, which subsequently correlates with increased vaccine hesitancy [11]. A significant correlation between the rise in populist political movements and increased vaccine hesitancy was reported before the COVID-19 pandemic, attributed to a profound distrust in elites and experts by disenfranchised groups [12].

Conspiracy theories, particularly those pertaining to health and vaccines, have gained significant traction in recent years, and are often propagated through social media platforms and alternative information sources [13,14]. In South Tyrol, the prevalence of such theories has been associated with the region’s distinctive sociocultural context, including its linguistic composition and historical experiences [10]. The dissemination of misinformation and beliefs in conspiracies has the potential to undermine public trust in vaccination programmes, consequently leading to reduced vaccination rates and the increased susceptibility to vaccine-preventable diseases.

Analysing the interplay between political factors, conspiracy beliefs, and vaccine hesitancy in South Tyrol is crucial for developing effective public health strategies. Comprehending the region’s sociocultural dynamics enables health authorities to design interventions that promote vaccine acceptance. This article examines the political influences and conspiracy theories contributing to vaccine hesitancy in South Tyrol with the aim of identifying its causes and proposing targeted strategies to enhance vaccine uptake.

## 2. The Sociopolitical Landscape of South Tyrol

### 2.1. Historical and Cultural Contexts

South Tyrol, an autonomous province in northern Italy, presents a unique sociocultural landscape influenced by its linguistic diversity. Approximately 70% of the population speak German as their mother tongue, 25% speak Italian, and 5% speak Ladin [15]. Linguistic groups have historically shaped health behaviours, including attitudes toward vaccine acceptance [16,17], through differing levels of trust in institutions and varying health literacy competencies. Studies suggest that German speakers in South Tyrol are less likely to trust national health authorities than Italian speakers [8], partially because of the historical tensions between the region and the Italian central government [18]. Beyond the established linguistic groups of German, Italian, and Ladin speakers, South Tyrol also hosts a growing immigrant population, categorised as ’others’ in vaccine studies [8]. Notably, this demographic exhibited higher levels of vaccine hesitancy compared to both German and Italian speakers across all age groups. Although specific studies on vaccine hesitancy within South Tyrol’s immigrant population are limited, the research consistently indicates that health-related decisions are shaped by cultural beliefs, attitudes, and values and that, in immigrant communities, these decisions may also be influenced by language barriers and varying levels of trust in the healthcare systems of their host countries [19,20,21]. For instance, mistrust and limited health literacy may impede vaccine acceptance [3], underscoring the need for tailored health communication strategies to address the unique challenges faced by immigrant populations.

Scepticism towards state-led initiatives, such as mandatory vaccinations [22], has impeded public health efforts in South Tyrol. The German-speaking population, culturally aligned with Austria, demonstrates a preference for Austrian healthcare practices in which childhood vaccinations are voluntary, in contrast to Italy’s mandatory policies [23]. This reliance on German-language Austrian healthcare information results in divergence in the adherence to health guidelines. Discrepancies between Italian and Austrian recommendations exacerbate mistrust towards the Italian system, engendering confusion and resistance. This misalignment has exacerbated the challenges of implementing public health measures in South Tyrol.

### 2.2. The COVID-19 Pandemic

The introduction of mandatory vaccination for healthcare workers during the COVID-19 pandemic has intensified the existing hesitancy [24]. During the COVID-19 pandemic, South Tyrol exercised its autonomy by implementing lockdown measures that differed in timing from national directives [25]. In March 2024, Italy’s Constitutional Court ruled that South Tyrol’s autonomous actions during the pandemic were unconstitutional, stating that the provincial legislature had overstepped its authority with the law enacted on 8 May 2020 [26]. However, regarding vaccination regulations, the province adhered to national policies without deviation. This approach led to criticism from groups advocating for personal freedom, who argued that while the region was willing to adjust lockdown measures, it did not extend the same flexibility to vaccination policies, thereby favouring economic considerations over individual rights [27,28]. In November 2021, the suspension of 111 physicians from a total of approximately 3100 in South Tyrol who declined COVID-19 vaccination elicited significant media attention and polarised public opinion [29]. This development underscored the challenges of enforcing public health measures in a region with deep-seated scepticism towards the state authority.

### 2.3. Political Dimension

The political landscape of South Tyrol has been fertile ground for parties promoting vaccine scepticism and conspiracy theories. A notable example is the “Süd-Tiroler Freiheit”, a regionalist and separatist political party advocating for the secession of South Tyrol from Italy and its reunification with Austria [30]. Founded in 2007, the party emphasised the preservation of the German-speaking identity and cultural heritage of the region. During the COVID-19 pandemic, “Süd-Tiroler Freiheit” has been associated with vaccine scepticism, questioning the efficacy and safety of vaccines, and opposing mandatory vaccination policies [28]. Notably, the “Süd-Tiroler Freiheit” [25] voiced opposition to mandatory vaccination measures.

In the 2023 post-pandemic elections, the political landscape shifted: the “Süd-Tiroler Freiheit” party gained additional seats in the regional council. Although it was critical for certain COVID-19 measures [31], its primary focus has shifted to other regional issues. Two new lists secured representation in the 2023 elections: the first was the “Vita” list, known for its strong opposition to COVID-19 vaccinations, which has been vocal in its criticism of vaccination mandates and other pandemic-related restrictions. The “Vita” party’s programme for South Tyrol emphasises the inviolability of human dignity and strongly opposes mandatory vaccinations, which are considered a violation of individual rights. The party advocates the protection of personal freedoms and fundamental human rights, particularly those that are individual and natural. This stance reflects a broader scepticism toward state-led health initiatives and aligns with narratives that question the legitimacy and safety of vaccination programmes [32]. The second party, the “Jürgen Wirth Anderlan (JWA)”, has been actively opposing COVID-19 vaccination policies, aligning with narratives that question the severity of the pandemic and the necessity of vaccination campaigns [33]. Both lists, Vita and JWA, continue to actively challenge vaccination efforts.

“Wir-Noi” is an additional organisation in South Tyrol that focuses on promoting regional autonomy and cultural identity [34]. In addition to these objectives, the group has been active in expressing scepticism toward vaccination policies, particularly during the COVID-19 pandemic. This stance is characterised by a critical view of mandatory vaccination measures, which they perceive as infringements on personal freedoms and individual rights. Their activities include organising protests and disseminating information that questions vaccine safety and efficacy. This approach aligns with broader vaccine hesitancy movements that emphasise personal autonomy and express distrust toward government-led health initiatives [34].

In summary, South Tyrol’s unique historical, cultural, and political context has created a complex environment to address COVID-19 vaccine hesitancy. Understanding these dynamics is crucial for developing tailored interventions that respect regional identity while promoting public health.

## 3. Typical Vaccine-Related Conspiracy Theories

### 3.1. Definition and Characteristics

Vaccine-related conspiracy theories frequently function as mechanisms for systemic critique, targeting public health institutions and government mandates [3]. These theories exploit distrust in authorities and present vaccines as deleterious, inefficacious, or components of broader nefarious agendas. From a psychological perspective, they provide a sense of comprehension and control during crises while socially reinforcing in-group identity among individuals with shared beliefs [4].

Such theories flourish in polarised environments, where misinformation is amplified by social media and influential figures [35]. They frequently overlap with narratives that stress individual autonomy and resistance to perceived overreach by the state or global organisations, undermining evidence-based public health measures [36].

### 3.2. Examples and Rebuttals

#### 3.2.1. “Vaccines Are Ineffective”

A prominent claim is that vaccines do not protect against diseases, which is often supported by cherry-picked data or anecdotal evidence. However, scientific consensus has robustly refuted this [37,38,39].

Vaccines, such as measles and rubella, have significantly reduced morbidity and mortality rates worldwide. For example, the WHO reports that the measles vaccine alone has prevented over 25 million deaths since 2000 [40]. Similarly, rubella vaccination campaigns have nearly eradicated congenital rubella syndrome in many regions [41]. This success is supported by the data from large-scale studies and surveillance programmes monitored by entities such as the European Centre for Disease Prevention and Control (ECDC) [42].

#### 3.2.2. “Vaccines Do More Harm than Good”

Another pervasive myth is that vaccines are inherently dangerous to humans. Claims like “the measles vaccine causes autism” persist despite being debunked. This narrative gained traction from Wakefield’s discredited study, which falsely linked the measles, mumps, and rubella (MMR) vaccine to autism [43].

Comprehensive research, including a landmark 2019 study involving over 650,000 children, demonstrated no association between vaccines and autism [44]. Leading health organisations, such as the CDC, WHO, and independent reviews, consistently affirm vaccine safety, emphasising that adverse events are exceedingly rare and typically outweighed by the benefits of immunisation [45,46].

#### 3.2.3. “The Pandemic Is a Planned Event”

Conspiracy theories pertaining to COVID-19 vaccines, particularly those invoking the “Great Reset” narrative, posit that the pandemic was orchestrated to impose global control [47]. The BBC reports that such unfounded theories allege that global elites planned the pandemic to reshape economies and societies to their advantage. These assertions distort legitimate discussions on post-pandemic recovery to engender fear and resistance. The World Economic Forum’s “Great Reset” initiative, introduced in June 2020, aims to address global challenges such as inequality and climate change through sustainable economic reforms [48]. However, proponents of conspiracy theories have misrepresented this agenda, suggesting that it serves as a blueprint for totalitarian world order. These unfounded claims exploit public uncertainty during crises, undermining the trust in public health measures and diverting attention from constructive policy debates.

Analyses of these narratives highlight their reliance on misinterpreted documents and speculative connections [49]. Experts from institutions such as the Lancet COVID-19 Commission and WHO underscore that such theories distract from urgent health priorities and exacerbate public distrust [50].

### 3.3. Vaccine-Related Conspiracy Theories in a Local Context

In South Tyrol, the organisation “WIR-NOI” [34], closely connected to right-wing and science-sceptical parties, illustrates how local cultural contexts can amplify vaccine-related conspiracy theories and disseminate them to the public. Their materials, including flyers and brochures, assert that childhood diseases provide “natural immune training” and posit that vaccines are not only unnecessary but also detrimental. Specific claims include the hypothesis that vaccines surreptitiously contaminate populations via blood transfusions or cause long-term health detriments, exploiting concerns regarding overreach by global health organisations, such as the WHO and pharmaceutical companies (Supplementary Figure S1). Additional conspiracy theories propagated by groups like “WIR-NOI” include the following:

- Vaccines as Tools for Population Control: the belief that vaccines are designed to reduce fertility or intentionally harm certain populations.
- Microchipping via Vaccination: the unfounded claim that vaccines contain microchips to track individuals.
- mRNA Vaccines Alter DNA: the incorrect assertion that mRNA vaccines modify human DNA.
- Vaccines Cause Severe Diseases: the false claim that vaccines lead to conditions such as cancer or autoimmune diseases.
- Shedding of Vaccine Components: the baseless idea that vaccinated individuals can “shed” vaccine components, affecting the unvaccinated population.

These conspiracy theories have been addressed and debunked by various fact-checking organisations. For instance, the PolitiFact refuted claims that COVID-19 vaccines cause cancer, noting that multiple studies have shown that vaccines save lives and do not cause such diseases [49]. Addressing conspiracy theories requires evidence-based communication, community engagement, and transparency to rebuild trust and promote vaccine acceptance.

The topic remains politically potent, drawing votes and public attention. This was evident in a recent press briefing organised by “Vita” party representatives, which emphasised ongoing critiques of the regional authorities’ approach to the COVID-19 vaccination rollout [51]. The party asserted that the government has failed to conduct an adequate review of the vaccination rollout and continues to disseminate misinformation to the public while utilising taxpayer funds to promote vaccines. Particular emphasis was placed on the administration of mRNA vaccines to pregnant women, asserting that these substances traverse the placental barrier and potentially pose risks to the foetuses [51]. During this event, various healthcare professionals corroborated these claims, reiterating concerns regarding the safety of mRNA vaccines. They posited that these vaccines could potentially lead to autoimmune diseases, myocarditis, and increased cancer incidence. A video presented at the conference further reinforced these assertions, portraying them as verified truths over time and advocating for a cessation of what was characterised as a “genetic experiment”.

Furthermore, the press conference featured a remote presentation by Dr. Maurizio Federico, a researcher affiliated with the Italian government, Istituto Superiore della Sanità (ISS) [52]. During this event, Dr. Federico emphasised the theoretical risks associated with mRNA vaccines, including the potential for the systemic distribution of vaccine components and the unregulated production of spike proteins in the body. Dr Federico published a “Perspective” article in the journal “Vaccines”, which focuses on the need for further research and proposing mucosal vaccines as a potentially safer alternative [53]. While the article raises valid scientific questions and suggests areas for improvement in vaccine technology, it does not draw broad conclusions or make alarmist claims as were presented during press conferences.

These claims demonstrate how conspiracy theories and selective interpretations of scientific discourse can influence public sentiment and intensify vaccine hesitancy in regions, such as South Tyrol. Such narratives exploit local concerns regarding autonomy and scepticism towards centralised health policies, further polarising the population, and undermining public health initiatives.

## 4. Discussion

Vaccine hesitancy in South Tyrol exemplifies the intricate interplay of historical, cultural, political, and socioeconomic factors that influence public perceptions of vaccination. This perspective article elucidates how these elements converge to perpetuate the scepticism regarding vaccines, propagate conspiracy theories, and impede public health initiatives. To enhance vaccine acceptance, it is essential to move beyond descriptive analyses and focus on targeted policy recommendations.

### 4.1. The Role of Historical and Cultural Dynamics

Across multilingual and diverse regions, vaccine hesitancy often stems from the mistrust in centralised authorities and the reliance on alternative health narratives. In South Tyrol, the partial preference for Austrian healthcare models and German-language health sources contribute to the lower confidence in Italian vaccination policies. This is mirrored in other European contexts, like Switzerland and Belgium, where cultural and linguistic divides influence vaccine attitudes [54,55,56]. Learning from these examples, South Tyrol’s health authorities should prioritise multilingual outreach and community-based interventions. The partial preference for Austrian healthcare practices and information in the German-speaking community often results in the conflicting adherence to Italian health policies. This divergence exacerbates mistrust and complicates the implementation of uniform vaccination campaigns. Addressing these dynamics necessitates public health authorities to develop communication strategies that acknowledge and respect cultural nuances.

In South Tyrol, cultural factors like the belief in natural immunity and traditional health practices reinforce vaccine hesitancy [9]. The perception that a healthy lifestyle—outdoor living, organic diets, and low pollutant exposure—reduces the need for vaccination complicates public health messaging. Acknowledging these ingrained cultural attitudes is essential for developing effective, culturally sensitive vaccination campaigns.

### 4.2. The Impact of Political Narratives

Vaccine hesitancy in Europe is shaped by disinformation campaigns that exploit political and regional identities, particularly in France, where scepticism is linked to populism and distrust in elites and institutions [57]. Hesitancy often aligns with populist movements that undermine trust in experts, reinforcing anti-vaccine sentiments [12,58]. In France, appeals to national sovereignty fuel scepticism, while in Italy, the misinformation about vaccines and autism contributed to declining immunisation rates [59]. Similar trends are observed across Europe, where disinformation campaigns exploit political and cultural divisions. In Poland, hesitancy aligns with right-wing populist narratives resisting perceived external control, reflecting broader anti-Western sentiments [60].

Similarly, in South Tyrol, local political parties have challenged national health directives. Studies of regions with comparable political landscapes suggest an effective approach to boost vaccine acceptance: depoliticising vaccine communication. This involves reframing the narrative from a government-led initiative to a community-driven health measure.

### 4.3. Conspiracy Theories and Misinformation

Conspiracy theories in South Tyrol, as in other regions, undermine confidence in vaccines by exploiting concerns regarding governmental overreach and perceived threats to individual autonomy [36]. Vaccine-related conspiracy theories are not unique to South Tyrol but have been observed globally. Studies from Germany [61] and the Netherlands [62] highlight the role of social media in amplifying these narratives, often linking them to a broader distrust in global health institutions. Research underscores that vaccine hesitancy often stems from the distrust in public institutions and scientific authorities, as observed in Austria, where sceptics framed the resistance to COVID-19 measures within their personal worldviews and cultural narratives [63]. These narratives not only erode public trust in vaccination programmes but also present considerable obstacles to achieving high immunisation rates. Addressing these theories requires targeted interventions that combine evidence-based refutation with culturally sensitive community engagement. Comparative evidence suggests that integrating counter-narratives into trusted local information channels, such as community radio or religious networks, can be more effective than centralised debunking efforts [64].

In South Tyrol, similar dynamics are evident, as linguistic and cultural diversity amplifies the scepticism toward state-led initiatives, requiring culturally sensitive interventions to rebuild trust and promote public health.

Vaccine scepticism is driven by disinformation and the reliance on alternative knowledge sources, especially among German-speaking communities. Many individuals prefer Austrian and German-language health information over Italian public health messaging, reinforcing doubts about national vaccination policies. This preference reflects historical and cultural ties, where trust is placed in sources aligned with local values. Addressing vaccine hesitancy requires engaging with these knowledge systems rather than just countering misinformation.

### 4.4. Bridging Scientific Discourse and Public Perception

Scientific findings are frequently subjected to selective interpretation and manipulation by the media and political actors to align with specific agendas. Although scientific articles may raise legitimate questions and propose alternative approaches, their nuanced discussions may be exaggerated or misrepresented in public forums, as evidenced by recent examples [51,53]. This phenomenon highlights a recurring issue: the oversimplification or sensationalisation of scientific findings to serve narratives that prioritise attention-grabbing headlines or political objectives over factual accuracy.

This highlights the challenges in knowledge transfer, a complex task often overlooked by institutions and challenging for scientists. Effective scientific communication requires specific skills that are not always possessed by the researchers. When unclear or poorly targeted, the public relies on the media, which can spread distorted or misleading narratives, increasing confusion and mistrust.

### 4.5. Critical Reflection on Science and Public Trust

While science serves as the foundation of public health advancements, it is not impervious to challenges, such as fraudulent practices, economic influences, and ethical transgressions. High-profile instances of scientific misconduct [65] and industry-driven agendas [66] occasionally undermine public confidence in research and its outcome. Acknowledging these limitations is imperative to fostering a transparent and accountable scientific community.

Nevertheless, it is equally crucial to emphasise the substantial body of evidence supporting key public health achievements. For instance, the eradication of smallpox and near-eradication of polio exemplify the transformative impact of vaccination programmes based on rigorous scientific principles [67]. Similarly, the significant decline in mortality from diseases such as diphtheria underscores the efficacy of evidence-based interventions [68].

Critical science, grounded in transparency, peer review, and replication, continues to function as a robust mechanism for distinguishing facts from conjecture. By openly addressing past shortcomings while demonstrating the proven success of public health initiatives, the scientific community can re-establish and maintain public trust. This dual approach reinforces the notion that science is self-correcting and inherently strives for the collective benefits of society.

### 4.6. Broader Lessons for Vaccine Acceptance

The situation in South Tyrol serves as a paradigmatic case for comprehending vaccine hesitancy in other regions characterised by strong cultural identities and historical scepticism towards centralised authority. The insights garnered from this context can inform global strategies for addressing vaccine hesitancy by emphasising the significance of contextualised approaches that balance the respect for local autonomy with the promotion of evidence-based health practices.

Public health measures frequently entail the implementation of regulations and policies that may limit individual freedom. However, these measures are predicated on the principle of safeguarding collective health and well-being. The examples include speed limits to reduce traffic-related injuries [69]; smoking prohibitions in public spaces to prevent second-hand smoke exposure [70]; and standards for air quality, water safety, and food hygiene to protect against environmental and dietary risks [71,72]. Unlike these widely accepted measures, vaccination regulations often face resistance, as they are perceived by some as infringing personal autonomy.

While such policies may be perceived as restrictive, they also function as critical instruments to mitigate preventable harm and promote societal health. The Italian experience demonstrates that mandating SARS-CoV-2 vaccination for healthcare workers significantly increased vaccination rates and decreased infection rates [73]. The balance between individual autonomy and public responsibility remains a recurring theme in health governance [74]. However, mandatory vaccinations differ significantly from other public health mandates in terms of public perceptions. Unlike measures such as smoking prohibitions, which provide an immediate and apparent benefit to individuals without a perceived risk, vaccination requires individuals to accept a potential personal risk (e.g., rare side effects) for the sake of collective benefit. This distinction underscores the necessity for tailored approaches to vaccine communication that address the unique challenges posed by the perceived trade-off between individual risks and societal gain [75].

Elucidating these parallels in public communication can facilitate the contextualisation of vaccination mandates as part of a broader effort to uphold societal health standards, potentially reducing resistance and fostering public trust in health interventions.

Individual liberty is the cornerstone of democratic societies that enables personal autonomy and decision-making. However, in public health, liberty is not absolute and must be balanced against community well-being. Mandatory vaccinations and smoking bans in public spaces are necessary to protect vulnerable populations and prevent harm. These examples illustrate balancing individual freedoms with societal responsibilities but are limited in scope. Emphasising the direct personal benefits of vaccination, such as the protection against severe diseases, is crucial to public acceptance. These measures follow the principle of proportionality, ensuring that they are no more restrictive than is necessary [76]. Negative freedom, or freedom from interference, must be balanced with positive freedom and the ability to act meaningfully within societal frameworks [77]. This approach resonates with the idea that freedom is not merely the absence of constraints but also the presence of enabling conditions for all members of society. Transparent communication of the policy rationale fosters trust and highlights the goal of protecting individual rights and the collective good.

### 4.7. Policy Implications and Recommendations

To effectively address vaccine hesitancy in South Tyrol, public health strategies must consider its unique sociocultural, linguistic, and political landscapes. The recommendations outlined in Table 1 emphasise the necessity for diverse approaches that balance respect for local diversity with evidence-based public health communications.

To enhance vaccine acceptance, recognising the role of credible communicators in public health messaging is crucial. In regions like South Tyrol, where the trust in central health authorities is limited, individuals often rely on local figures such as community doctors and alternative health practitioners for medical advice. These trusted messengers can bridge the gap between scientific recommendations and public concerns by framing vaccine information to align with cultural and health beliefs.

Culturally sensitive communication is essential in regions where linguistic and cultural diversity influences health behaviours. Campaigns that align with local values and address the specific concerns of key demographics, such as young adults and parents, are more likely to engender trust and engagement. Community engagement strategies can utilise the credibility of trusted local figures, such as healthcare professionals and community leaders, to bridge the gap between public health authorities and sceptical populations.

Transparency and accessibility in public health communication are critical. Openly acknowledging vaccine risks and proactively disseminating safety and efficacy data can mitigate concerns and foster confidence in vaccination programmes. Additionally, the rapid dissemination of misinformation on social media necessitates robust monitoring and regulation to prevent the proliferation of conspiracy theories. By implementing these strategies in a coordinated and adaptive manner, public health authorities can address the underlying causes of vaccine hesitancy, rebuild trust, and promote a higher vaccination uptake in South Tyrol.

These recommendations serve not only as a framework for South Tyrol, but also as a model for addressing vaccine hesitancy in other regions with similarly complex sociocultural dynamics.

### 4.8. Limitations

This study draws on a targeted literature review, primarily informed by prior research on vaccine hesitancy in South Tyrol [5] and supplemented by additional peer-reviewed and grey literature. While this approach provides focused insights into the political and sociocultural dimensions of vaccine hesitancy, it is not a systematic review and may introduce selection bias.

The reliance on region-specific sources limits the study’s broader applicability, as vaccine attitudes vary across contexts. Although comparative insights from other European regions were included, they remain selective rather than comprehensive.

This study focuses on conspiracy narratives and political influences as contributors to vaccine hesitancy, potentially overemphasising misinformation-driven hesitancy. Vaccine attitudes are shaped by multiple factors that warrant further exploration to complement the political and media-driven aspects discussed.

## 5. Conclusions

The examination of vaccine hesitancy in South Tyrol reveals a complex interplay between historical conflicts, cultural identities, political influences, and misinformation. The region’s unique linguistic and cultural landscape necessitates tailored public health strategies that respect local contexts, while addressing vaccine concerns and conspiracy theories. The key lessons from this case study include the need for culturally appropriate communication, community involvement, and transparency in addressing vaccine concerns. The active monitoring and control of misinformation, especially on social media, is crucial for countering unfounded fears and restoring trust in public health authorities.

The selective misinterpretation of scientific discussions highlights the importance of clear and accessible communication of evidence-based health information to reduce the impact of alarmistic narratives. Bridging the gap between subtle scientific debates and public understanding is essential for mitigating the effects of misinformation. By integrating this understanding into public health strategies, decision-makers and healthcare professionals can enhance vaccine confidence, increase vaccine uptake, and protect communities from preventable diseases. The broader implications of this study extend to other regions with complex sociocultural dynamics, emphasising the global importance of context-specific approaches to overcome vaccine hesitancy.

Public health initiatives aim to cultivate informed, critical, and health-literate citizens. Through the provision of accurate information and the development of evaluative skills, individuals become active participants in their own health and contribute to the collective resilience against misinformation.

## Figures and Tables

**Table 1:** Strategies to address vaccine hesitancy in South Tyrol.

Strategy	Description
Culturally Sensitive Communication [5]	Campaigns should be tailored to South Tyrol’s linguistic and cultural diversity, emphasising local values while debunking misinformation. Additionally, they should focus on age groups, particularly young adults and parents, who are key decision-makers for the next generation’s health.
Community Engagement [78,79]	Collaborating with trusted local figures, such as healthcare professionals and community leaders, can enhance credibility and foster trust.
Transparency and Accessibility [80]	Open acknowledgment of vaccine risks, coupled with the proactive dissemination of safety data, can build public confidence.
Monitoring and Regulation of Misinformation [14,81]	Strengthening the surveillance of misinformation, particularly on social media, is crucial to curbing the spread of conspiracy theories.

## Data Availability

No new data were created.

## Supplementary Materials

The following supporting information can be downloaded at: https://www.mdpi.com/article/10.3390/ijerph22020230/s1, Figure S1: English translation of the WIR-NOI brochure.
