# Supplementary material for: The Interplay of Politics and Conspiracy Theories in Shaping Vaccine Hesitancy in a Diverse Cultural Setting in Italy
Source: Int J Environ Res Public Health. 2025 Feb 6;22(2):230. doi: 10.3390/ijerph22020230 (PMC11855398; doi:10.3390/ijerph22020230)
Supplement: Supplementary file 1 [file ijerph-22-00230-s001.zip › ijerph-3442333-supplementary.pdf]

Supplementary Material

Figure S1. English Translation of the WIR-NOI Brochure. This figure presents a translated version of the WIR-NOI brochure distributed in South Tyrol, which exemplifies vaccine-related conspiracy narratives. The original document, written in German and Italian, contains claims regarding the safety and efficacy of vaccines, including assertions about vaccine-related health risks, alleged global health agendas, and alternative views on public health measures. The translation aims to provide insight into the rhetoric and themes used to promote vaccine hesitancy in the region. The content has been translated using using DeepL Translator (www.deepl.com, Cologne, Germany). The original brochure was produced and disseminated publicly by the WIR-NOI organization. The use of this material in an academic publication falls under fair use principles for educational and research purposes, as it is provided for the purpose of critique, analysis, and public health discourse. However, any additional reproduction or distribution of this translation should acknowledge the original creators and may require explicit permission from WIR-NOI.

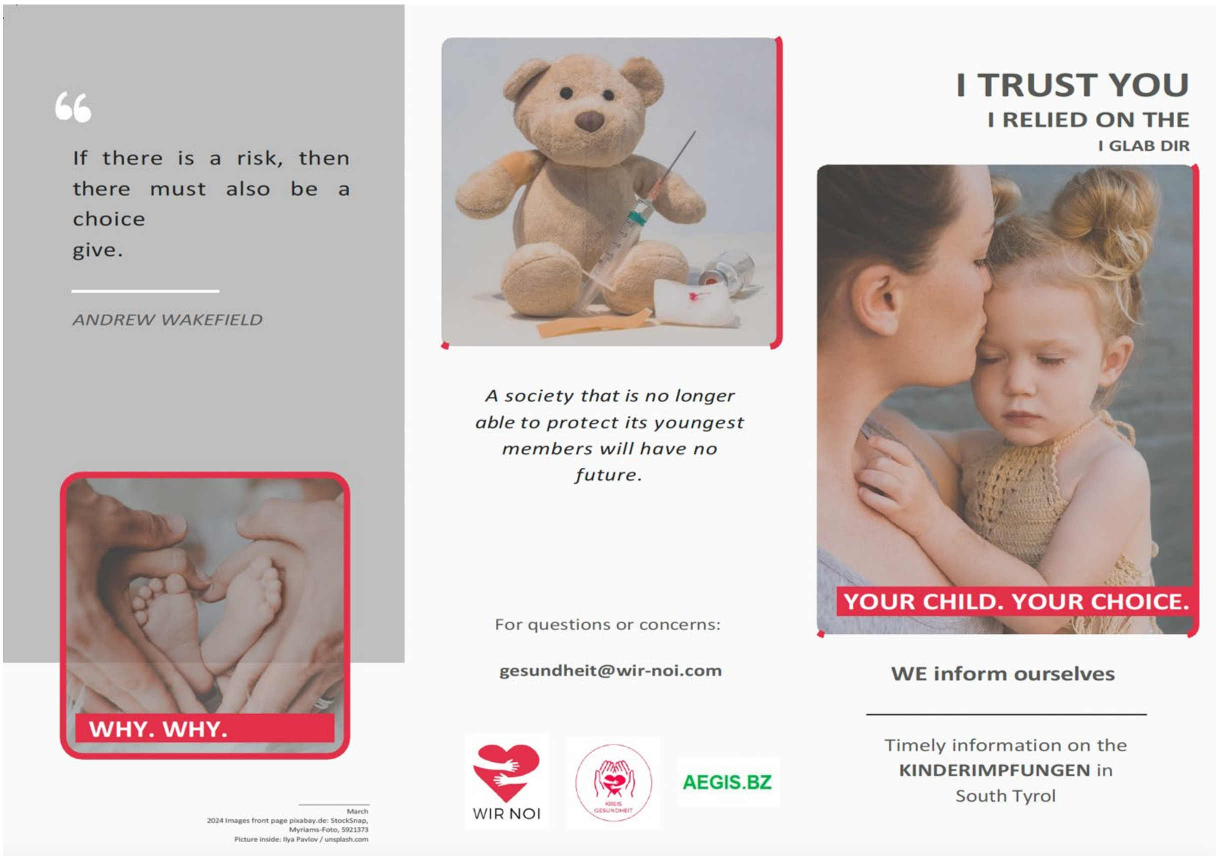

DID YOU KNOW THAT ... ?

- ... **childhood illnesses** are **training for the immune system** and children who have been through these illnesses are healthier.
- ... Vaccination **does not prevent a disease** protects. *Cohort studies*
- ... Children up to the age of 3 have not developed a complete **myelin sheath** around the nerves and the blood-brain barrier is still open and therefore every **vaccination is an attack on the nervous system**.
- ... especially childhood illnesses have a **biological purpose** and are even beneficial for children's development. *Dr Ryke Geerd Hamer, MD*
- ... Vaccinations contain numerous **toxic additives,** such as thiomersal (mercury derivative), aluminium hydroxide, formaldehyde and foreign proteins.
- ... every vaccination harbours **considerable risks** and numerous contraindications appear on the individual package leaflets (please read).
- ... **autoimmune diseases** can be triggered by th vaccinations.
- ... in particular the **measles vaccination** is responsible for the **exponential rise in autism** in children. *Documentary Vaxxed*
- ... healthy children who do not fulfil the state vaccination schedule are not allowed to attend childcare or kindergarten (prerequisite: **10 compulsory vaccinations** according to the Lorenzin 119 law of 2017).

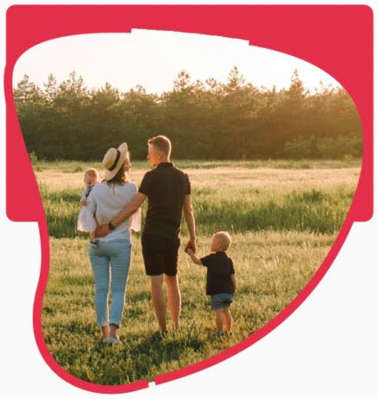

BOOKS & DOCUS

Books:

- The vaccination decision. Dr Friedrich P. Graf, 2023
- Turtles all the way down: Science and The myth of vaccination. Mary Holland J.D., 2023
- What you always wanted to know about vaccination. Bert Ehgartner, 2023
- Vaccinated sick - those affected tell us. Dr Carola med. Javid-Kistel, Rolf Korn, Ulrike Gerstmayer, 2020
- Health from a single mould. Dr Jürgen Birmanns, 2019
- Various vaccination guides. Ravi Roy & Carola Lage-Roy, 1990 - 2024
- Homeopathy for everyone. Dr Johann Loibner, 2017
- Vaccination. The facts. Wolfram Klingele, 2016
- Childhood illnesses, yesterday-today-tomorrow. Daniel Trappitsch, 2014
- Life without vaccination, testimonials. Andreas Bachmair, 2012

Documentaries:

- We do not vaccinate. Michael Leitner
- Under the skin. Bert Ehgartner
- Vaxxed 1+2. Andrew Wakefields 2017 + 2020
- Invisibili: Il film sugli effetti collaterali dei vaccini anti Covid. 2022

DID YOU KNOW THAT ... ?

- ... From 0 to 6 years of age, **32 combined compulsory vaccine doses** are administered in 6 injections (the additional "recommended vaccinations" are not counted here).
- ... the lever of **compulsory vaccination** is tantamount **to blackmail** and parents should not put up with it.
- ... a right (**right to education**) cannot be cancelled out by an obligation (**compulsory vaccination**).
- ... **South Tyrol** could separate compulsory vaccination from compulsory education **due to its autonomy** and yet there are training courses that require compulsory vaccination.
- ... all health data falls under the **law of privacy** and parents cannot be obliged to pass on sensitive data.
- ... the WHO plans to produce all vaccines on **an mRNA basis.** *Georg Dellapietra - Safeblood Donation*
- in a **blood transfusion**, the recipient is immunised through the back door. *Georg Dellapietra - Safeblood Donation*
- ... the **decline in infectious diseases** cannot be attributed to vaccination. *Dr Johann Loibner, MD*
- ... children who have already been vaccinated should undergo **alternative medical treatment**.
- ... every injection is a **breach of trust** in the child's parents. *Dr Ryke Geerd Hamer, MD*
